# Case Report: Selective PRP application during staged MEEK-based reconstruction of extensive deep burns

**DOI:** 10.3389/fsurg.2026.1921231

**Published:** 2026-10-21

**Authors:** Baoxiang Tian, Xue Ren, Hailong Lin, Yang Liu, Chunlin Wei, Dujuan Liu, Fucheng Zhang, Xiong Yang

**Affiliations:** Department of Burn and Plastic Surgery, Jilin Chemical Hospital, Genertec Baoshihua Medical, Jilin City, Jilin Province, China

**Keywords:** burns, case report, meek micrografting, platelet-rich plasma, staged reconstruction, wound closure

## Abstract

**Background:**

Staged grafting may be required after extensive deep burns when donor skin is limited. We describe the clinical course of three patients treated with selective platelet-rich plasma (PRP) application during MEEK-based reconstruction.

**Case description:**

Three men with 21%, 45%, and 40% total body surface area (TBSA) burns underwent staged debridement and autologous grafting. PRP was administered during two of two, three of four, and one of three burn-wound operations, respectively. Approximate, chart-recorded graft-take estimates ranged from 80% to 95% across procedures, including procedures without PRP; these estimates were not obtained using a documented standardized measurement method. At discharge, Patient 1 had complete epithelialization, whereas Patients 2 and 3 had small residual wounds. At photographic follow-up approximately 2.5–3.5 months after discharge, no recurrent deep burn wounds or clinically documented infection were noted; Patient 1 had superficial scar ulcerations. Retrospectively reported Vancouver Scar Scale (VSS) scores were 8, 9, and 10, with incomplete documentation of the original assessment method.

**Conclusion:**

The cases describe an individualized staged-reconstruction pathway incorporating selectively applied PRP. They do not establish a separate effect of PRP on graft take, infection, healing, or scar outcomes.

## 1 Introduction

Extensive deep burns often require serial excision and grafting because early wound closure must be balanced against limited donor skin and systemic instability (1–3). MEEK micrografting increases the area covered by a split-thickness graft through expansion of small skin islands (4, 5). Comparative evidence pertains to different clinical settings and cannot be generalized to patients with large, medically complex burns (6).

PRP has been studied as an adjunct to burn wound care and split-thickness grafting, but preparations, modes of administration, and clinical outcomes vary across studies (7, 8). We report three patients treated with individualized debridement, selective PRP wound-bed administration, and MEEK or other autologous grafting. The purpose is to describe clinical decision-making and observed short-term outcomes, not to estimate the independent efficacy of PRP.

## 2 Case descriptions

### 2.1 Patient 1

History and initial assessment. A 39-year-old man was admitted on 13 July 2025, one day after sulfuric acid exposure. He had first received water irrigation, topical wound care and dressings elsewhere. No chronic disease or long-term medication was documented. The face, trunk, upper and lower limbs, and feet were affected; total burn area was approximately 21% TBSA, including 15% full-thickness and 5% partial-thickness injury (the remaining depth was not specified). Leathery, insensate areas supported the clinical diagnosis of full-thickness burn. No clinical wound infection was documented initially, and a wound culture yielded no growth.

Treatment and course. On 15 July, the wounds were debrided and tangentially excised. Scalp split-thickness skin (approximately 3% TBSA) was prepared as 25 MEEK sheets at 1:4 expansion to cover approximately 12% TBSA; approximately 5 mL of autologous PRP was administered locally to the recipient wound bed before graft placement. Recorded graft take was approximately 85% on postoperative day (POD) 7. On 23 July, six further MEEK sheets from approximately 1% TBSA of scalp skin covered approximately 4% TBSA of residual face, trunk, and limb wounds, again with PRP; recorded graft take was approximately 90% on POD10. Serial dressings, nanosilver dressings, topical epidermal growth factor, and scar-care measures (10) accompanied surgery. Unhealed wounds were recorded as approximately 3%–4% TBSA on 1 August and approximately 1% TBSA on 13 August. Complete epithelialization was documented at discharge on 7 September, 56 days after admission (Figure 1; Supplementary Tables 1 and 2).

**Figure 1:**
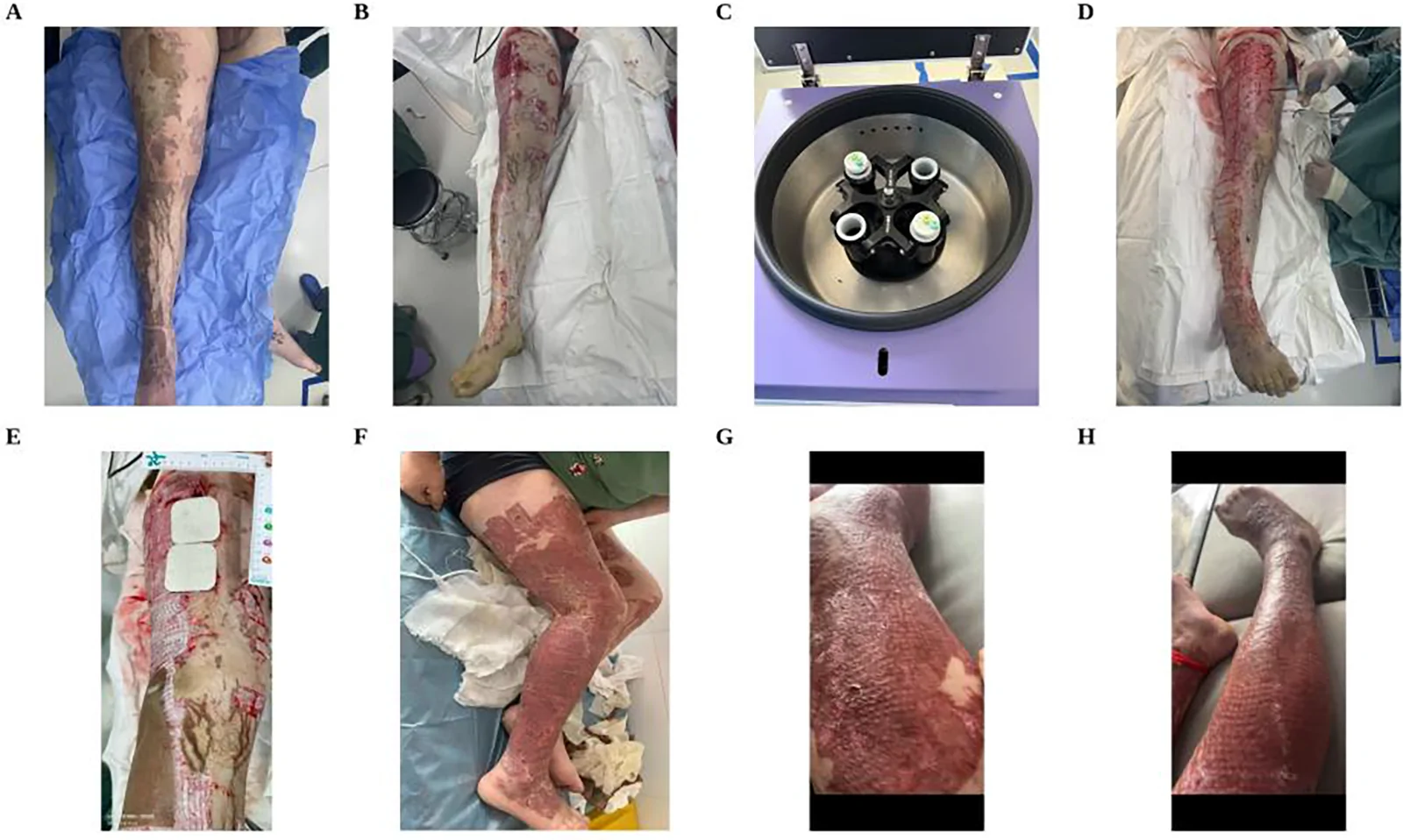
Patient 1: staged reconstruction and clinical follow-up. **(A)**: Initial lower-limb chemical burn (13 July 2025). **(B)**: After debridement and tangential excision (15 July). **(C)**: PRP preparation (15 July). **(D)**: Local PRP microdroplet administration (15 July). **(E)**: MEEK graft placement (23 July). **(F)**: Wound closure near discharge (7 September). **(G)** and **(H)**: Scar appearance with small superficial ulcerations at follow-up (15 December 2025).

### 2.2 Patient 2

History and initial assessment. A 60-year-old man was admitted on 12 September 2025, approximately 3 h after flame injury, without documented professional prehospital treatment. Burns involved the head, face, neck, trunk and limbs (45% TBSA: approximately 30% full thickness, 12% deep partial thickness, with additional more superficial areas). He had untreated hypertension and mild inhalation injury. Initial care included oxygen, inhalation therapy, resuscitation, dressing changes, transfusion as required, analgesia, and blood-pressure control. A 14 September wound culture grew Enterobacter cloacae, reported susceptible to meropenem and piperacillin/tazobactam and resistant to first-generation cephalosporins. The combination of this result and local purulent discharge was consistent with wound infection. Intravenous meropenem 1 g every 8 h was recorded for 14 days.

Treatment and course. Four burn-wound operations took place on 15, 18 and 24 September and 10 October. At the first operation, scattered eschar and thin purulent exudate were removed; the post-excision wound bed was red and actively bleeding without visible residual necrosis. Once resuscitation was completed and the patient was circulatorily stable, the surgical team proceeded with grafting rather than leaving the large excised area open. Approximately 13% TBSA was covered using 26 MEEK sheets (1:4), alongside additional split-thickness grafting and xenogeneic acellular dermal matrix coverage. Further operations treated the limbs, trunk and residual wounds; PRP was administered during the first three but not the final stamp-grafting procedure. Recorded graft take ranged from approximately 80% (first operation, POD7) to 95% (last operation without PRP, POD10). A repeat wound culture was reported negative on 28 September. A separate inpatient summary mentions an unspecified Gram-negative culture finding around 1 October; the sample details and its relationship to the earlier culture could not be established from the available records. A right lower-limb intramuscular venous thrombosis developed and was treated with anticoagulation. At discharge on 21 October, after approximately 39 days, only small granulating wounds remained, requiring outpatient dressings (Figure 2; Supplementary Tables 1 and 2).

**Figure 2:**
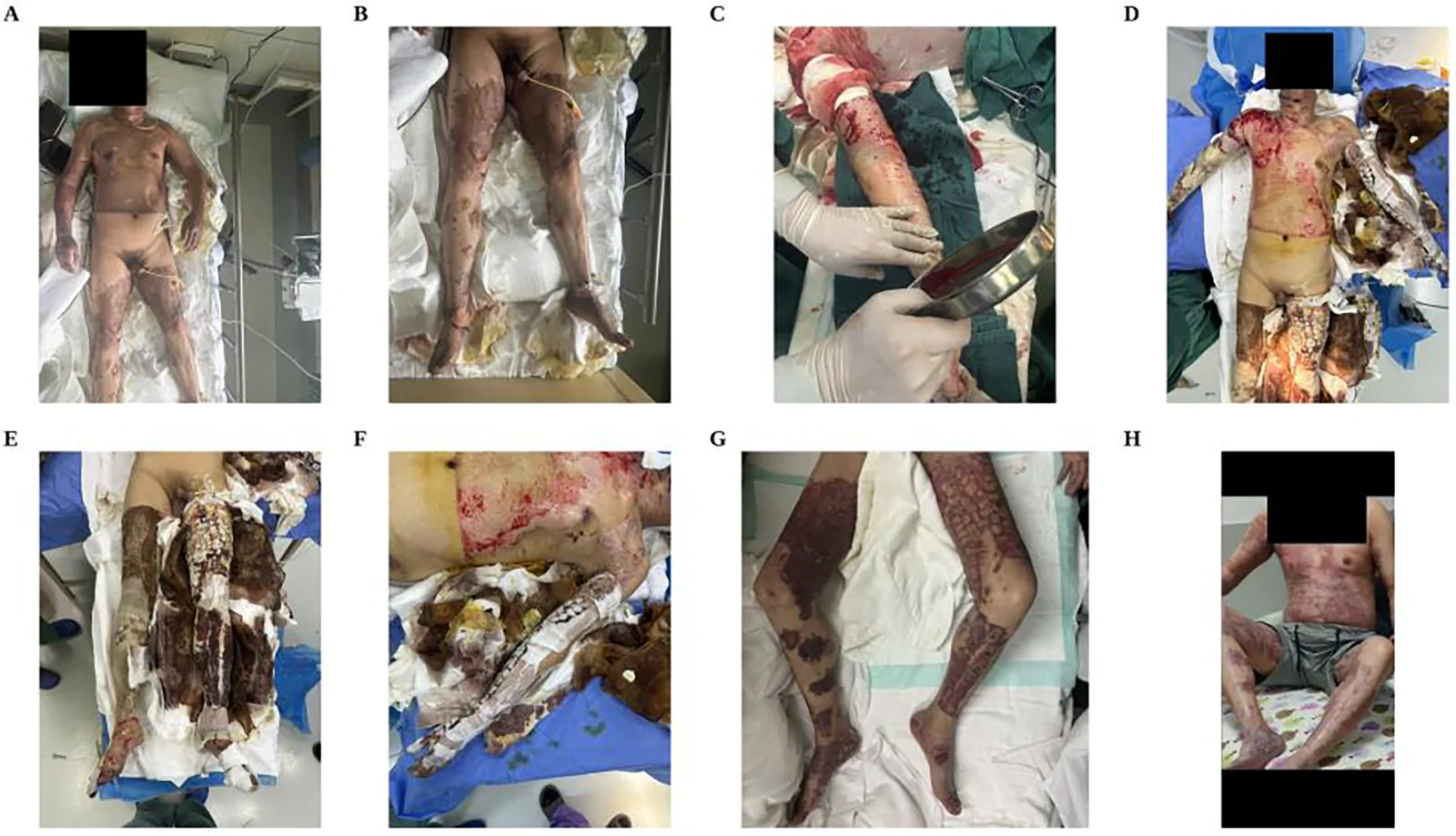
Patient 2: staged wound treatment and follow-up. **(A)** and **(B)**: Initial flame burns (12 September 2025). **(C)**: First-stage debridement (15 September). **(D)**: Grafting and temporary coverage (15 September). **(E)** and **(F)**: Early postoperative wound condition and further surgical care (18 September). **(G)**: Later-stage residual-wound care. **(H)**: Scar appearance at follow-up (25 January 2026).

### 2.3 Patient 3

History and initial assessment. A 57-year-old man was admitted on 27 September 2025, two days after flame injury involving the left upper limb, trunk, lower limbs and buttocks (40% TBSA, including approximately 35% full-thickness and 5% deep partial-thickness burns). Mild inhalation injury was also reported in the clinical history. He had type 2 diabetes of more than 10 years’ duration, previously treated with oral acarbose, coronary artery disease and acute coronary syndrome. Initial laboratory records described leukocytosis, anemia, hyperglycemia, low albumin and low calcium, without retained quantitative values in the supplied case summary. The 28 September wound culture grew Acinetobacter baumannii/calcoaceticus complex and an Enterococcus species. Together with necrotic wounds, purulent discharge and the documented systemic clinical course, the treating team recorded wound infection and burn sepsis; the available records do not provide a complete retrospective organ-dysfunction assessment to independently apply formal sepsis criteria. The isolates were reported susceptible to meropenem/polymyxin and linezolid, respectively. Intravenous meropenem 1 g every 8 h and linezolid 0.6 g every 12 h were documented for 21 days.

Treatment and course. Following resuscitation and assessment of operative tolerance, debridement and tangential excision on 29 September removed gray-yellow necrotic tissue and focal purulence, leaving a viable, bleeding wound bed. Scalp-derived skin was expanded at 1:4 to produce 23 MEEK sheets; approximately 5 mL PRP was administered locally before graft placement, and vacuum sealing drainage was applied. Recorded graft take was approximately 82% on POD7. On 2 October, coronary angiography identified severe multivessel coronary stenosis; the patient declined intervention and continued medical treatment. Additional MEEK grafting on 9 October and stamp grafting on 23 October were performed without PRP; charted graft-take estimates were 89% and 93%, respectively. Repeat wound culture on 19 October was reported negative, and the treating team documented clinical control of infection. At discharge on 1 November, 35 days after admission, approximately 1% TBSA of granulating wounds remained for outpatient care (Figure 3; Supplementary Tables 1 and 2).

**Figure 3:**
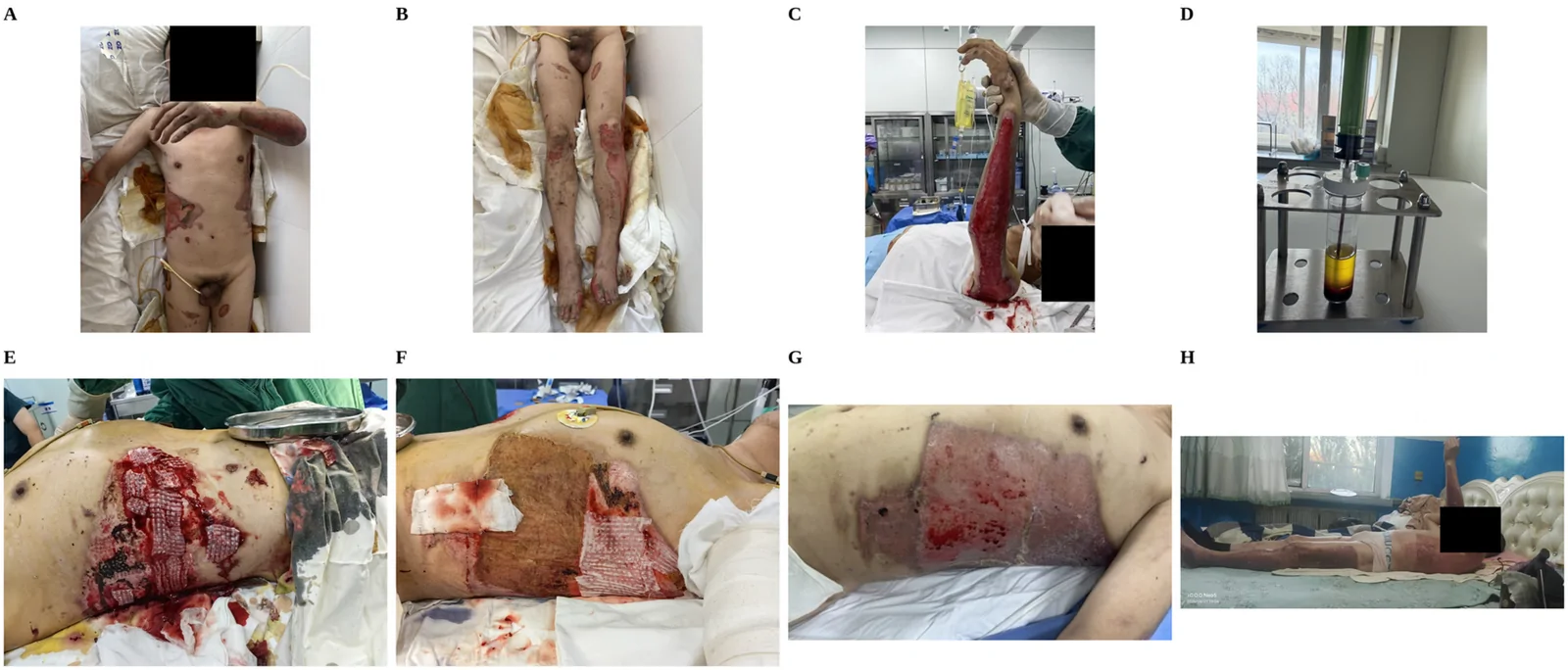
Patient 3: wound treatment during a complicated burn admission. **(A)** and **(B)**: Initial burns (27 September 2025). **(C)**: Debridement (29 September). **(D)**: PRP preparation (29 September). **(E)**: MEEK grafting (29 September). **(F)** and **(G)**: Postoperative and later-stage wound appearance. **(H)**: Scar appearance at follow-up (15 January 2026).

### 2.4 Clinical timeline

Key dated clinical events are summarized in Table 1; more detailed chronological timelines for each patient, including microbiology, burn-wound interventions, PRP exposure, discharge and follow-up, are presented in Supplementary Table 2. The three patients underwent two, four and three burn-wound operations, respectively; the separate coronary angiography in Patient 3 is not counted as a burn-wound operation.

**Table 1:** Baseline characteristics, key clinical timeline, treatment and outcomes.

Variable	Patient 1	Patient 2	Patient 3
Age/sex	39 years/male	60 years/male	57 years/male
Admission	13 Jul 2025; 1 day after injury	12 Sep 2025; ∼3 h after injury	27 Sep 2025; 2 days after injury
Cause/total area	Sulfuric acid; 21% TBSA	Flame; 45% TBSA	Flame; 40% TBSA
Burn depth	∼15% full thickness; ∼5% partial thickness	∼30% full thickness; ∼12% deep partial thickness; remainder not quantified	∼35% full thickness; 5% deep partial thickness
Major comorbidities	None documented	Grade 3 hypertension; mild inhalation injury	Diabetes; coronary disease/acute coronary syndrome; mild inhalation injury reported
Wound microbiology	Culture: no growth	E. cloacae; repeat culture reported negative	A. baumannii/calcoaceticus complex and Enterococcus sp.; repeat culture reported negative
Burn-wound operations	2	4	3 (plus separate coronary angiography)
Operations with PRP	2/2	3/4	1/3
Initial culture/assessment	Wound culture: no growth (date unavailable)	14 Sep 2025: E. cloacae; clinical infection	28 Sep 2025: Acinetobacter complex and Enterococcus sp.
PRP-assisted surgery dates	15 and 23 Jul 2025	15, 18 and 24 Sep 2025	29 Sep 2025
Surgery dates without PRP	None	10 Oct 2025	9 and 23 Oct 2025
Other dated events	1 Aug: 3%–4% TBSA unhealed; 13 Aug: ∼1%	28 Sep: negative culture reported; ∼1 Oct: unspecified Gram-negative finding	2 Oct: coronary angiography; 19 Oct: negative culture reported
Discharge/closure	7 Sep; fully epithelialized	21 Oct; small residual granulating wounds	1 Nov; ∼1% TBSA residual granulating wound
Dated follow-up	15 Dec 2025	25 Jan 2026	15 Jan 2026
Retrospective VSS total	8	9	10
Clinical follow-up	Hypertrophic scars; small superficial ulcerations; no obvious ROM limitation documented	Stable scars; thrombosis reportedly resolved; no obvious ROM limitation documented	Stable scars; no obvious ROM limitation documented

PRP, platelet-rich plasma; ROM, range of motion; TBSA, total body surface area; VSS, Vancouver Scar Scale.

Graft-take estimates were approximate and are detailed in Supplementary Table 1. Follow-up intervals differed among patients.

## 3 Therapeutic intervention and outcome assessment

After clinical assessment of burn depth and recipient-bed viability, excision and debridement were performed until grossly nonviable tissue had been removed and a bleeding recipient bed was obtained. Split-thickness skin was harvested mainly from the scalp; thigh or lower-leg sites were also used in Patient 2. MEEK grafts were cut into approximately 3-mm islands and expanded at 1:4 (4–6). Stamp-like grafts, xenogeneic acellular dermal matrix and vacuum sealing drainage were used at selected stages. Antimicrobial treatment, dressing care, nutritional and fluid support, comorbidity management and rehabilitation were provided as indicated (1, 2, 9).

According to the documented PRP preparation protocol, autologous blood (30 mL for Patient 1; 40 mL for Patients 2 and 3) was collected using 3.2% sodium citrate (blood:anticoagulant 9:1). Centrifugation was reported at 1,800 × g for 10 min at room temperature, collection of plasma with the buffy-coat interface, followed by 2,400 × g for 10 min; a portion of platelet-poor plasma was removed and the lower fraction retained. No exogenous calcium chloride or thrombin was added. Approximately 5 mL of product per PRP-assisted procedure was administered as superficial microdroplets locally to the recipient wound bed before grafting, using a 1-mL syringe and 25-gauge blunt needle. The precise surface area receiving PRP was not recorded; this volume should not be interpreted as coverage of the entire grafted area. The clinical summary indicates that post-preparation platelet and leukocyte counts were measured, but the corresponding numerical results, fold enrichment and yield were not retained in the available case records; no cellular PRP subtype can therefore be assigned.

PRP was not applied according to a prespecified dosing schedule: it was used in both of Patient 1's operations, the first three of Patient 2's four operations, and only the first of Patient 3's three burn-wound operations. The records do not establish a quantitative rule for selecting the exact PRP-treated surface or withholding PRP at later stages. The selected intervention was one component of multimodal reconstruction, not an isolated exposure.

The percentages labelled “graft take” in the records were approximate clinical estimates at POD7 or POD10 for the respective operations (Supplementary Table 1). The retrospective documentation does not specify the assessor, a uniform denominator, calibrated planimetry, or an adjudicated protocol; some procedures combined MEEK with other graft or coverage methods. Accordingly, the values are reported descriptively and are neither directly comparable across stages nor suitable for PRP-specific efficacy calculations. Discharge healing was described as complete epithelialization or near-complete closure with residual granulating wounds. No standardized prospective functional testing or patient-reported outcome instrument was documented.

## 4 Follow-up and outcomes

Patient 1 was discharged with complete epithelialization; Patients 2 and 3 had small residual areas that required outpatient dressings. Available follow-up observations were dated 15 December 2025, 25 January 2026 and 15 January 2026, respectively, corresponding to approximately 2.5–3.5 months after discharge. No recurrent extensive burn wound or clinically documented infection was reported at these visits. Patient 1 had hypertrophic scars with small superficial ulcerations, whereas Patients 2 and 3 had stable scars without reported ulceration. The available summaries described no obvious joint-motion limitation or contracture, but no goniometric range-of-motion results were provided. The right lower-limb thrombosis in Patient 2 was reported resolved following anticoagulation, and Patient 3's diabetes and cardiac condition remained clinically stable.

Retrospectively reported VSS totals were 8, 9 and 10 (vascularity/pigmentation/pliability/height: 2/2/2/2, 2/2/3/2 and 3/2/3/2; standard component ranges are 0–3, 0–2, 0–5 and 0–3, respectively). The available retrospective records did not identify the original scorer, standardized anatomical assessment site, or contemporaneous palpation protocol for pliability. These values describe the reported short-term scars but are not independently verified or suitable for between-patient comparison. No longer-term mature-scar assessment was available.

## 5 Discussion

These three cases document staged reconstruction for extensive deep burns in patients with different wound causes and systemic risks. MEEK expansion and additional grafting permitted progressive coverage while debridement, antimicrobials, dressings and supportive care addressed other clinical problems. The experience shows how selective PRP application was incorporated into an individualized surgical pathway, but does not demonstrate that PRP improved any measured outcome.

PRP was used in six of nine burn-wound operations and was omitted from later procedures in Patients 2 and 3. Later procedures without PRP also had charted graft-take estimates. Such nonrandomized, different-stage observations cannot serve as within-patient controls: recipient-bed condition, graft technique and wound extent changed over time. Prior reviews have described heterogeneous PRP interventions and uncertain transferability across burn and grafting settings (7, 8). The absence of platelet/leukocyte counts and of a quantified treated area further limits product-level interpretation.

In Patients 2 and 3, grafting took place after removal of grossly necrotic and purulent tissue to a viable wound bed, together with antimicrobial treatment and assessment of systemic stability. The decision was made in the context of extensive full-thickness injury and risks associated with prolonged open wounds, not as a strategy for placing grafts onto uncontrolled infected tissue. The reported culture results and subsequent wound progress cannot isolate any effect of PRP or graft timing; in Patient 2, the microbiology summaries contain an unresolved difference between sampling dates.

The principal limitations are the three-case sample, lack of a comparator, different comorbidities and burn mechanisms, mixed graft and wound-care interventions, variable PRP exposure, unavailable product-cell counts, approximate graft-take estimates without a standardized assessment protocol, short and nonuniform follow-up intervals, retrospective VSS reporting, and lack of measured joint range of motion or authentic patient-reported perspectives. These shortcomings also prevent estimating an optimal PRP timing, dose, or patient subgroup. Prospective studies with characterized PRP, prespecified wound-area and graft-take assessment, comparators and longer functional and patient-reported follow-up are needed.

## 6 Patient perspective

Direct patient-reported perspectives were not available for this retrospective case series. No first-person patient accounts or formal patient-reported outcome measures were collected in the available follow-up records. The observations on scar care and functional status in Section 4 were documented by the treating clinicians and do not represent the patients’ own experiences. No patient statements or patient-reported outcomes were retrospectively reconstructed.

## 7 Conclusion

In three patients with extensive deep burns, staged MEEK-based reconstruction incorporated PRP at selected operations and was followed by complete or near-complete discharge wound closure. The cases provide procedural and short-term clinical observations but do not establish an independent benefit of PRP over MEEK-based reconstruction without PRP.

## Data Availability

The original contributions presented in the study are included in the article/Supplementary Material, further inquiries can be directed to the corresponding author.
