# Supplementary material for: Case Report: Selective PRP application during staged MEEK-based reconstruction of extensive deep burns
Source: Front Surg. 2026 Oct 21;13:1921231. doi: 10.3389/fsurg.2026.1921231 (PMC13731286; doi:10.3389/fsurg.2026.1921231)
Supplement: Supplementary file 1 [file Table1.docx]

Supplementary Table 1. Detailed burn-wound operations, PRP use and reported graft take

| **Patient** | **Date** | **Recipient site / area** | **Donor site / area** | **Graft technique** | **PRP** | **Wound-bed status / graft take** |
| --- | --- | --- | --- | --- | --- | --- |
| Patient 1 | 15 Jul 2025 | Limbs; MEEK ~12% TBSA | Scalp ~3% TBSA | MEEK 1:4; 25 sheets | Yes (~5 mL) | Viable bed after debridement/tangential excision; POD7 graft take ~85% |
| Patient 1 | 23 Jul 2025 | Residual face/trunk/limb wounds ~4% TBSA | Scalp ~1% TBSA | MEEK 1:4; 6 sheets | Yes (~5 mL) | Residual wound bed debrided; POD10 graft take ~90% |
| Patient 2 | 15 Sep 2025 | Limbs/trunk; MEEK ~13% TBSA; xenogeneic coverage ~25% TBSA | Scalp ~3% + right thigh ~3% TBSA | MEEK 1:4; 26 sheets; additional graft/dermal coverage | Yes (~5 mL) | Thin purulent exudate/eschar before debridement; viable bleeding bed after complete debridement; POD7 graft take ~80% |
| Patient 2 | 18 Sep 2025 | Left upper/lower limbs ~26% TBSA; trunk coverage ~13% TBSA | Left thigh ~10% TBSA | MEEK/split-thickness grafting; xenogeneic dermal coverage | Yes (~5 mL) | Scant fibrinous exudate, no obvious purulence; red well-perfused bed after debridement; POD10 graft take ~88% |
| Patient 2 | 24 Sep 2025 | Trunk ~13% TBSA | Lower leg ~5% TBSA | Stamp-like split-thickness grafting | Yes (~5 mL) | Aged granulation without purulence; refreshed to well-bleeding base; POD7 graft take ~92% |
| Patient 2 | 10 Oct 2025 | Scattered residual wounds ~10% TBSA | Scalp ~3% TBSA | Stamp-like split-thickness grafting | No | Small granulating wounds without necrosis/purulence; POD10 graft take ~95% |
| Patient 3 | 29 Sep 2025 | Trunk, bilateral lower limbs, buttocks | Scalp | MEEK 1:4; 23 sheets; VSD | Yes (~5 mL) | Necrotic tissue with focal scant purulence before debridement; viable bleeding bed after removal; POD7 graft take ~82% |
| Patient 3 | 9 Oct 2025 | Residual left upper-limb wound | Scalp | MEEK 1:4; 10 sheets; VSD | No | Edematous granulation, no substantial purulence; refreshed to bleeding base; POD10 graft take ~89% |
| Patient 3 | 23 Oct 2025 | Scattered residual wounds ~10% TBSA | Scalp | Stamp-like split-thickness grafting | No | Granulating wounds without necrosis/purulent exudate; POD7 graft take ~93% |

Graft-take percentages are approximate values transcribed from routine clinical records at POD7 or POD10. The original records do not document a standardized denominator, independent assessor or calibrated planimetry. Some operations used mixed techniques and temporary coverage; the listed percentages cannot be interpreted as MEEK-specific outcomes or as a comparison of PRP-exposed and unexposed sites. PRP volume was recorded as approximately 5 mL per PRP-assisted procedure and was not documented as distributed over the entire recipient area.

Abbreviations: MEEK, modified Meek micrografting; POD, postoperative day; PRP, platelet-rich plasma; TBSA, total body surface area; VSD, vacuum sealing drainage.

Supplementary Table 2. Clinical timelines for three burn patients

| **Patient** | **Date** | **Recorded event / outcome** | **PRP** |
| --- | --- | --- | --- |
| Patient 1 | 13 Jul 2025 | Admission: chemical burn, 21% TBSA; prior water irrigation and local treatment | — |
| Patient 1 | 15 Jul 2025 | Debridement, tangential excision and MEEK 1:4 (25 sheets) | Yes (~5 mL) |
| Patient 1 | 22 Jul 2025 | POD7 after first MEEK: reported graft take ~85% | — |
| Patient 1 | 23 Jul 2025 | Repeat debridement and MEEK 1:4 (6 sheets) | Yes (~5 mL) |
| Patient 1 | 1 Aug 2025 | Unhealed wound area recorded as ~3–4% TBSA | — |
| Patient 1 | 2 Aug 2025 | POD10 after second MEEK: reported graft take ~90% | — |
| Patient 1 | 13 Aug 2025 | Unhealed wound area recorded as ~1% TBSA | — |
| Patient 1 | 7 Sep 2025 | Discharge: documented complete epithelialization; 56 days after admission | — |
| Patient 1 | 15 Dec 2025 | Dated scar follow-up: hypertrophic scars and small superficial ulcerations; retrospectively reported VSS 8 | — |
| Patient 2 | 12 Sep 2025 | Admission: flame burns, 45% TBSA; hypertension and mild inhalation injury | — |
| Patient 2 | 14 Sep 2025 | Wound culture: Enterobacter cloacae; meropenem treatment reported | — |
| Patient 2 | 15 Sep 2025 | Debridement; MEEK 1:4 (26 sheets), other grafting and temporary coverage | Yes (~5 mL) |
| Patient 2 | 18 Sep 2025 | Further limb grafting and trunk temporary coverage | Yes (~5 mL) |
| Patient 2 | 22 Sep 2025 | POD7 after 15 Sep procedure: reported graft take ~80% | — |
| Patient 2 | 24 Sep 2025 | Trunk stamp-like grafting | Yes (~5 mL) |
| Patient 2 | 28 Sep 2025 | POD10 after 18 Sep procedure: reported take ~88%; repeat wound culture reported negative | — |
| Patient 2 | ~1 Oct 2025 | An inpatient summary also mentions an unspecified Gram-negative wound-culture finding; sample details could not be reconciled | — |
| Patient 2 | 1 Oct 2025 | POD7 after 24 Sep procedure: reported graft take ~92% | — |
| Patient 2 | 10 Oct 2025 | Residual-wound stamp grafting; no PRP | No |
| Patient 2 | 20 Oct 2025 | POD10 after 10 Oct procedure: reported graft take ~95% | — |
| Patient 2 | 21 Oct 2025 | Discharge: small residual granulating wounds; 39 days after admission; venous thrombosis treated during admission | — |
| Patient 2 | 25 Jan 2026 | Dated scar follow-up: no recurrent infection; VSS 9 retrospectively reported; thrombosis reportedly resolved | — |
| Patient 3 | 27 Sep 2025 | Admission: flame burns, 40% TBSA; diabetes and coronary disease | — |
| Patient 3 | 28 Sep 2025 | Wound culture: Acinetobacter complex and Enterococcus species; wound infection/burn sepsis recorded by treating team | — |
| Patient 3 | 29 Sep 2025 | Debridement, MEEK 1:4 (23 sheets) and vacuum sealing drainage | Yes (~5 mL) |
| Patient 3 | 2 Oct 2025 | Coronary angiography: severe multivessel disease; intervention declined (not a burn-wound operation) | — |
| Patient 3 | 6 Oct 2025 | POD7 after first MEEK: reported graft take ~82% | — |
| Patient 3 | 9 Oct 2025 | Further MEEK 1:4 (10 sheets), vacuum sealing drainage; no PRP | No |
| Patient 3 | 19 Oct 2025 | POD10 after 9 Oct procedure: reported graft take ~89%; repeat wound culture negative | — |
| Patient 3 | 23 Oct 2025 | Residual-wound stamp grafting; no PRP | No |
| Patient 3 | 30 Oct 2025 | POD7 after 23 Oct procedure: reported graft take ~93% | — |
| Patient 3 | 1 Nov 2025 | Discharge: ~1% TBSA residual granulating wound; 35 days after admission | — |
| Patient 3 | 15 Jan 2026 | Dated scar follow-up: no recurrent infection reported; VSS 10 retrospectively reported | — |

Notes: Operative dates and their postoperative graft-take assessment dates are shown separately. POD-specific estimates come from clinical records without a documented standardized assessment protocol. Patient 2’s 28 September negative culture and a separate summary indicating an unspecified Gram-negative finding around 1 October could not be reconciled; neither constitutes proof of PRP-mediated infection control. Follow-up dates represent nonuniform intervals after discharge. The time of venous thrombosis onset was not available.
