# Supplementary material for: Case Report: Selective PRP application during staged MEEK-based reconstruction of extensive deep burns
Source: Front Surg. 2026 Oct 21;13:1921231. doi: 10.3389/fsurg.2026.1921231 (PMC13731286; doi:10.3389/fsurg.2026.1921231)
Supplement: Supplementary file 2 [file Table2.docx]

CARE reporting checklist

Manuscript: Case Report: Selective PRP Application During Staged MEEK-Based Reconstruction of Extensive Deep Burns

Checklist status reflects the available three-case clinical records. “Not available” identifies an absent source item rather than a result reconstructed from photographs or other indirect evidence.

| Item | CARE element | What is reported / missing | Manuscript location | Status |
| --- | --- | --- | --- | --- |
| 1 | Title | “Case Report” appears in the manuscript title. | Title | Reported |
| 2 | Keywords | Principal clinical topic, technique, adjunct and article type. | Keywords | Reported |
| 3a | Abstract: introduction | Brief rationale for staged wound closure and PRP use. | Abstract, Background | Reported |
| 3b | Abstract: case presentation | Burn extent, interventions and outcomes for three patients. | Abstract, Case description | Reported |
| 3c | Abstract: conclusion | Limited to descriptive feasibility; no PRP-specific efficacy claim. | Abstract, Conclusion | Reported |
| 4 | Introduction | Clinical context and published literature. | Section 1 | Reported |
| 5a | Patient information | Age, sex, injury type and relevant history. | Sections 2.1–2.3; Table 1 | Reported |
| 5b | Symptoms / primary concerns | Burn location and clinical presentation; formal patient-stated chief complaint not retained. | Sections 2.1–2.3 | Partially reported |
| 5c | Medical / family / psychosocial history | Available comorbidities, prior treatment and medications; other domains not documented. | Sections 2.1–2.3 | Partially reported |
| 6 | Clinical findings | Burn area, depth and available wound-bed and clinical features. | Sections 2.1–2.3; Table 1 | Reported |
| 7 | Timeline | Dated summary plus expanded chronological sequence, including assessment dates. | Section 2.4; Table 1; Supplementary Table 2 | Reported |
| 8a | Diagnostic methods | Clinical burn examination, cultures, coronary angiography, reported laboratory findings. | Sections 2.1–2.3 | Reported |
| 8b | Diagnostic challenges | Retrospective absence of some quantitative values; ambiguous Patient 2 culture sequence. | Sections 2.2–2.3; Section 5 | Reported |
| 8c | Diagnostic reasoning | Burn depth, infection and viability assessment; Patient 3 sepsis recorded by team without full criteria ascertainment. | Sections 2.1–2.3; Section 5 | Reported with limitations |
| 8d | Prognostic / staging | Burn extent and depth; systemic comorbidities. | Sections 2.1–2.3; Table 1 | Reported |
| 9a | Intervention types | Debridement, MEEK and other grafts, PRP, adjunctive wound and systemic care. | Sections 2.1–2.3; Section 3; Supplementary Table 1 | Reported |
| 9b | Administration / dosing | Available PRP protocol, blood draw, volumes, route and antimicrobials. PRP cell counts not available. | Section 3; Supplementary Table 1 | Partially reported |
| 9c | Intervention changes | Stages with versus without PRP documented; no prespecified selection rule recorded. | Section 3; Supplementary Table 1 | Reported with limitations |
| 10a | Clinician/patient outcomes | Discharge healing, short-term scars and nonquantified movement; no patient-reported outcomes. | Section 4; Table 1 | Partially reported |
| 10b | Follow-up tests | Dated follow-up and selected microbiology; conflicting Patient 2 result explicitly noted. | Sections 2.2–2.3; Section 4; Supplementary Table 2 | Reported with limitations |
| 10c | Adherence / tolerability | Routine care documented; systematic adherence or tolerability not recorded. | Sections 2–4 | Partially reported |
| 10d | Adverse / unanticipated events | Venous thrombosis, residual wound and superficial scar ulceration. | Sections 2.1–2.3; Section 4; Table 1 | Reported |
| 11a | Discussion: strengths and limitations | Three patients, no comparator, heterogeneous care and incomplete measurements. | Section 5 | Reported |
| 11b | Discussion: literature | MEEK and PRP evidence cited. | Sections 1 and 5; References | Reported |
| 11c | Discussion: causal reasoning | No PRP-specific effect attributed. | Section 5 | Reported |
| 11d | Take-away conclusion | Short-term clinical course and limits of inference. | Section 7 | Reported |
| 12 | Patient perspective | No first-person patient perspective or patient-reported outcome available; clinician notes were not substituted. | Section 6; Section 4 (clinical follow-up) | Not available |
| 13 | Informed consent | Written informed consent for publication of case details and accompanying clinical images is reported in the Ethics statement. | Ethics statement | Reported |

This checklist is for transparent reporting. A missing patient perspective is not represented as fulfilled by consent for publication or clinician follow-up observations.
